# Annotations

**Published:** 1905-11-04

**Authors:** 


### ANNOTATIONS.

### The British Science Guild.

The inaugural meeting of the British Science Guild, which took place on Monday last at the Mansion House, may be regarded as an event of the first importance. The Guild, which was founded in the spring of last year, differs from the Royal Society and the British Association in having for its object, not the promotion of scientific knowledge for its own sake, but the general recognition of its overwhelming value and the advantage of employing the methods of scientific inquiry in affairs of every kind. That there is a very promising field for such a society is not to be doubted, and if its efforts succeed to the satisfaction of its promoters, among whom are the leaders of scientific thought to-day, the result may be no less a thing than a fresh lease of prosperity for the people of the British Empire. As Sir Norman Lockyer said in the course of his address to the meeting, those studies which embrace ancient civilisations and their literatures are by themselves as incapable of forming the complete man as would be the mere facts of science apart from the actual use of the methods of observation and discovery. A complete education must be based upon things and thinking, as well as upon words and memory. What is wanted is an education which will not merely teach people to know, but will also teach them to think. The Western world has stood agape at the recent display of Japanese efficiency. But the explanation of the efficiency is simply this, that during the last thirty years every Japanese child has been taught how to think, and, as an inevitable consequence, how to create. Hitherto the nations of the West have owed their superiority to their practical monopoly of creative thought. The monopoly is theirs no longer. To hold their own in the future it will be necessary to cultivate with care the creative faculty which is present in some degree in every child, instead of leaving it, as has been the custom in the past, to thrive or wither away as chance may decide. This is the work which the British Science Guild has before it, and we hope they may achieve substantial success by their endeavours.

### Christian Science in Practice.

An example of the folly of putting faith in the treatment of Christian Science practitioners is afforded by the case of a school-teacher on whose remains a coroner's inquest was held on Saturday. The deceased, a young man of twenty-eight, was for some time an assistant master at a college in Hull. Early in October the Principal of the institution wrote to his father in London to say that his son was in a very serious condition of health and would not consult a medical man, as he had adopted Christian Science teaching. Subsequently the latter came to London, and, in spite of the protest of his father, placed himself under the care of a Christian Science practitioner at Richmond, at a charge of ten shillings a week. Eventually a doctor was sent for, but found the young man dead when he arrived. The Christian Science practitioner, Mr. Harold Boardman, who is also by profession a school-teacher, was sworn, and said that he did not believe in drugs, and considered "that God was the only doctor." He had treated the deceased according to his views. As to these views, the jury, after hearing from the medical man in attendance that the life of the deceased could at any rate have been prolonged by proper treatment, returned a verdict of death from natural causes, but censured Mr. Boardman for not sending for a doctor. With regard to faith-healing which has thus so disastrously failed, Mr. Swayne, we observe, has repudiated our suggestion that he is coquetting with the movement. But while he usefully mentions, as disposing of the idea that Christian Scientists are exempt from the ordinary ills of life, the circumstance that two of them recently apologised for breaking an engagement with him because they were suffering from measles, he says that it is not wise for the clergy to condemn Christian Science unreservedly. We do not see how it can be wise to refrain from condemning a system under which it is inevitable that human lives must run the risk of being needlessly sacrificed. If Mr. Boardman's patient had been a child of tender years he would certainly have discovered that Christian Science also involves serious consequences to those who venture to put its tenets into practice.

### A Courageous Chairman.

The Chairman of the Fulham Guardians has had the courage to express a fear that must be in the minds of many who note the trend of public action with regard to the relief of the poor. He has refused to serve on the Distress Committee of his parish, saying that from what he saw last year he is convinced that the idea of finding employment for the poor out of the rates is dangerous to all classes, not least to the poor themselves. This is the view that is taken by many, but in these days it is so unpopular that it is to a large extent held silently, while the thoughtless and the voluble, in their ignorant sympathy with the poor, try to help them in ways that serve only to perpetuate, if not to increase, their poverty. In strict logic, there is no such thing as "finding work for the unemployed." If there is no real necessity for the work it is, phrase it how one may, simply relief with a labour-test attached. Employment such as this will only make the men less active when they return, if they ever do, to genuine competitive labour. Still more dangerous is the suggestion that the unemployed might get work from private employers, and deficiency of wages be made up out of the rates. Such an expedient was proposed and tried in the early part of last century, with the result of reducing nearly the whole working class to practical pauperism, while astute employers found it cheaper to take into their service paupers whose insufficient wages were eked out by doles from the rates than to engage independent labourers. It is perhaps impossible to prevent the rates being the pension fund of the aged poor, but we shall enter upon a more dangerous development when we allow them to become the wage fund of the able-bodied.

